# The Long-Term Clinical Impact of Thoracic Endovascular Aortic Repair (TEVAR) for Advanced Esophageal Cancer Invading Aorta

**DOI:** 10.1245/s10434-021-10081-3

**Published:** 2021-06-03

**Authors:** Ke-Cheng Chen, I-Hui Wu, Chih-Yang Chang, Pei-Ming Huang, Mong-Wei Lin, Jang-Ming Lee

**Affiliations:** 1 Division of Thoracic Surgery, Department of Surgery, College of Medicine, National Taiwan University Hospital, Taipei, Taiwan; 2 Division of Cardiovascular Surgery, Department of Surgery, National Taiwan University Hospital and College of Medicine, Taipei, Taiwan

## Abstract

**Background:**

Advanced esophageal cancer invading the aorta is considered unsuitable for surgery with definitive chemotherapy or chemoradiation as the treatments of choice. In the current study, we evaluated the long-term clinical impact of combining thoracic endovascular aortic repair (TEVAR) with multimodality treatment in caring for such patients.

**Methods:**

We evaluated 48 patients who had advanced esophageal cancer with aortic invasion. The oncological outcome, including overall survival (OS) and progression-free survival (PFS), after multimodality treatment with or without TEVAR is evaluated for these patients.

**Results:**

Overall, 25/48 patients (52.1%) received a TEVAR procedure. There was no significant difference in OS (*p* = 0.223) between patients who did or did not receive TEVAR; however, patients who received TEVAR had significantly less local tumor recurrence (*p* = 0.020) and longer PFS (*p* = 0.019). This impact was most evident in patients who received both TEVAR and esophagectomy, with an incremental increase in hazard ratio (HR) for disease progression of 2.89 (95% confidence interval [CI] 0.86–9.96) and 4.37 (95% CI 1.33–14.33) observed under multivariable analysis, respectively, in comparison with patients who underwent only one or neither of these procedures (*p* = 0.005 for trend test).

**Conclusion:**

TEVAR is a feasible procedure for esophageal cancers invading the aorta and can be used for curative-intent resection to improve local tumor control and PFS.

**Supplementary Information:**

The online version contains supplementary material available at 10.1245/s10434-021-10081-3.

Esophageal cancer is one of the most fatal malignancies, primarily because of its aggressive nature and proximity to vital organs such as the trachea and aorta,^1^ with 5-year overall survival (OS) of 15–25%^2^ Advanced esophageal cancer with aortic invasion is classified as T4 disease according to the 8th American Joint Committee on Cancer (AJCC) staging system^3^ and the recommended treatment is definitive chemoradiation or chemotherapy without surgery (National Comprehensive Cancer Network [NCCN] guideline, version 3, 7 July 2020, 220). However, disease progression is frequently encountered after such treatment, with a median survival of 10.6 months, and 14.6% of patients end up with aortoesophageal (AE) fistulas during or after treatment.^4^

Esophagectomy in the setting of aortic mural invasion is conventionally contraindicated. Thoracic endovascular aortic repair (TEVAR) is an effective and less-invasive treatment for aortic aneurysms.^5^,^6^ Recently, some studies have applied this technique to prevent impending aortic rupture or treat AE fistulas associated with cancer invasion, chemoradiation therapy or esophagectomy.^7^–^9^ In patients with locally advanced esophageal cancer invading the aorta, some surgeons have even used TEVAR as a bridging therapy for subsequent salvage esophagectomy or chemoradiation.^10^ However, only short-term outcomes and feasibility were reported in the aforementioned studies regarding TEVAR as a combined therapy for esophageal cancer. This study reviews our experience of using TEVAR to treat esophageal cancers invading the aorta in combination with other treatment modalities and assesses both the short- and long-term outcomes.

## Methods

### Study Population

**Fig. 1:**
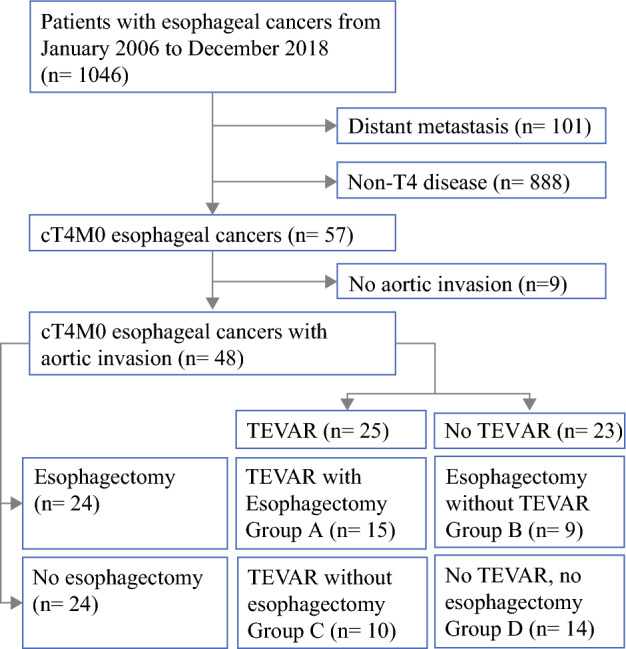
Study collection from the esophageal cancer patient cohort. *TEVAR* thoracic endovascular aortic repair

A total of 1046 patients with esophageal cancer treated in National Taiwan University Hospital from January 2006 to December 2018 were reviewed in this study. The inclusion criterion of this study was clinical T4 esophageal cancer with aortic invasion (based on the AJCC 8th edition).^3^ TEVAR was administered to patients with suspected bleeding from AE fistulas (emergency) or tumor invasion to the aorta determined by clinical studies. The definition of aortic invasion by tumor was based on computed tomography (CT) and positron emission tomography (PET) scans using the following criteria: (1) direct contact between the esophageal tumor and aorta of more than one-quarter the circumference of the aorta on any axial cut of contrast in the CT image; or (2) obliteration of the demarcation/tissue plane between the esophageal tumor and the aorta.^11^–^13^ We excluded esophageal cancer patients with distant metastasis or recurrent disease, resulting in the inclusion of 48 esophageal cancer patients with aortic invasion in this study (Fig. 1). The Research Ethics Committee of the hospital approved this retrospective study (202003044RINA) and waived informed consent.

For patients with confirmed esophageal cancer, we completed all staging surveys, including a contrast CT, endoscopic ultrasound, tumor biopsy, bronchoscopy, PET, pulmonary function test, echocardiogram, and barium meal study, before treatment. Both clinical and pathological staging for esophageal cancer were based on the 8th edition AJCC/Union for International Cancer Control (UICC) Cancer Staging Manual.^3^ For patients with T3 or N1 disease or above, neoadjuvant concurrent chemoradiation (CCRT) was performed, followed by surgery 4–8 weeks after CCRT or 9–13 weeks after TEVAR. Surgical methods of esophagectomy included McKeown and Ivor Lewis procedures, with two- or three-field lymph node dissections performed, and both procedures were performed using either a uni- or multiportal laparothoracoscopic technique after TEVAR.^14^ Frozen pathology was examined after resection for any suspected lesion, to ensure a free proximal and distal margin of the esophagus. OS and PFS were defined between the period of intervention with TEVAR and mortality, or clinical confirmation of disease progression or recurrence, respectively.

### Thoracic Endovascular Aortic Repair (TEVAR) Procedure

All procedures were performed 1–2 weeks before simulation of the radiation field and were conducted using a hybrid suite (Artis Zeego system, Siemens Healthcare, Forchheim, Germany) under general anesthesia with tracheal intubation and femoral cut-down or percutaneous access. In the prophylactic group, the treatment goal was to cover the site of aortic invasion adjacent to the esophageal cancer to prevent bleeding, while in the emergency group, the TEVAR procedure was performed to treat aortic hemorrhage. The location of the invasive lesion or hemorrhage was determined by preoperative CT and PET scan, measuring proximally from the left subclavian artery or distally to the celiac artery. If the patient’s condition was stable before TEVAR, the oral edge of the tumor was marked with a radiopaque metallic clip by esophagoscopy to mark the optimal site for the stent graft. The proximal landing zone was selected between zones 0 and 4, depending on the location of the esophageal cancer; proximal and distal sealing of at least 2 cm was required. The selected diameter size of the stent graft was 10–20% larger than the aortic diameter at the proximal landing zone. Tapered devices were selectively used in patients with smaller distal aortic landing zones. To achieve an adequate proximal landing zone, a chimney procedure or physician-modified fenestration was performed as needed in some patients and was rarely indicated for the distal landing zone. Routine spinal drainage was not required due to the short segment of aortic coverage or the emergency indications. Blood pressure was strictly controlled at 140/80 mmHg postoperatively to prevent spinal cord ischemia after TEVAR.

### Patient Follow-Up

Patients were followed-up at outpatient clinics by way of a physical examination, panendoscopy, and contrast CT (brain, neck, chest and abdomen), every 6 months. PET scan, bronchoscopy, or other examinations were performed whenever any symptom or sign indicating tumor progression or recurrence occurred. We defined local progression as tumor progression at the resection margin, anastomosis site, or original location, while regional progression was defined as newly noted abdominal, mediastinal, supraclavicular, or cervical lymphadenopathy, which was stated in terms of a lymph node larger than 1 cm in the short axis on CT. Distant metastasis was described exactly as a metastatic lesion in another organ. The progressing lesion was further confirmed by surgical biopsy, endobronchial ultrasound biopsy, or endoscopic biopsy if necessary and feasible.

### Statistical Analysis

In this study, descriptive statistics are reported as mean ± standard deviation, while categorical variables are reported as number and percentage. The normality of continuous variables was evaluated using the Kolmogorov–Smirnov test. We performed a *t*-test for continuous variables with a normal distribution, but otherwise we used the Mann–Whitney U test. A Pearson Chi-square test or Fisher’s exact test was performed for categorical variables. OS and PFS were calculated using the Kaplan–Meier method and analyzed using the log-rank test. A multivariable analysis was conducted using the Cox regression model, which reduced the significant confounding bias in the univariate analysis. Statistical Analysis System (SAS) version 9.4 software (SAS Institute Inc., Cary, NC, USA) was used for all analyses. A *p*-value <0.05 was considered significant.

## Results

### Patient Demographics, Clinical Features and Treatments

This study cohort included 48 patients who had locally advanced esophageal cancers invading the aorta. Twenty-five patients received TEVAR and 24 patients received esophagectomy (Fig. 1). The clinical profiles of patients treated with or without TEVAR are listed in Table 1. There was no statistical difference between the two groups of patients in T and N staging, neoadjuvant therapy, esophagectomy, or any other potential confounding variable aside from hypertension, which had a significantly higher incidence in the TEVAR group (36.0% vs. 8.7%, *p* = 0.024). In the TEVAR with esophagectomy group, a complete resection was achieved in 67% of patients (10/15), with 5 cases of tumor involvement in the circumferential margin (33%, 5/15). In addition, the disease progression rate was remarkably lower in patients treated with TEVAR than those not treated with TEVAR (28.0% vs. 69.6%, *p* = 0.020).

**Table 1:** Comparison of clinical characteristics and outcomes in patients treated with and without TEVAR

				Total [*n* = 48]		TEVAR [*n* = 25]		No TEVAR [*n* = 23]		*p*-value
Sex, male				45 (93.8)		25 (100)		20 (87.0)		0.062
Age, years				58.8 ± 8.5		60.2 ± 8.5		57.3 ± 8.4		0.229
ASA classification		1		0		0		0		0.128
		2		24 (50)		16 (64.0)		8 (34.8)		
		3		19 (39.6)		7 (28.0)		12 (52.2)		
		4		5 (10.4)		2 (8.0)		3 (13.0)		
		5		0		0		0		
Smoking				40 (83.3)		20 (80.0)		20 (87.0)		0.518
Drinking				32 (66.7)		14 (56)		18 (78.3)		0.102
Betelnut chewing				16 (33.3)		7 (28.0)		9 (39.1)		0.413
Comorbidity		CAD		2 (4.2)		1 (4.0)		1 (4.4)		0.952
		Liver cirrhosis		2 (4.2)		2 (8.0)		0		0.165
		CKD		1 (2.1)		1 (4.0)		0		0.332
		COPD		2 (4.2)		0		2 (8.7)		0.132
		DM		3 (6.3)		3 (12.0)		0		0.086
		Heart failure		1 (2.1)		0		1 (4.4)		0.292
		Hypertension		11 (22.9)		9 (36.0)		2 (8.7)		0.024
		Hyperlipidemia		1 (2.1)		1 (4.0)		0		0.332
		Others		16 (33.3)		10 (40.0)		6 (26.1)		0.307
Tumor location		Upper		8 (16.7)		4 (16.0)		4 (17.4)		0.819
		Middle		19 (39.6)		9 (36.0)		10 (43.5)		
		Lower		21 (43.8)		12 (48.0)		9 (39.1)		
Pathological N stage		pN0		12 (25.0)		7 (28.0)		5(21.7)		0.243
		pN1		9 (18.8)		5 (20.0)		4(17.4)		
		pN2		2 (4.2)		2 (8.0)		0		
		pN3		2 (4.2)		2 (8.0)		0		
		cNx (no surgery)		23 (47.9)		9 (36.0)		14 (60.9)		
Esophagectomy and reconstruction		No surgery		24 (50.0)		10 (40.0)		14 (60.9)		0.274
		Tri-incision		18 (37.5)		12 (48.0)		6 (26.1)		
		Ivor Lewis		6 (12.5)		3 (12.0)		3 (13.0)		
CRT		No		1 (2.1)		1 (4.0)		0		0.212
		Definite		19 (39.6)		6 (24.0)		13 (56.5)		
		Neoadjuvant		19 (39.6)		12 (48.0)		7 (30.4)		
		Adjuvant		3 (6.3)		2 (8.0)		1 (4.4)		
		Neoadjuvant + adjuvant		6 (12.5)		4 (16.0)		2 (8.7)		
Cell type		Squamous cell carcinoma		45 (93.8)		23 (92.0)		22 (95.7)		0.625
		Adenocarcinoma		2 (4.2)		1 (4.0)		1 (4.3)		
		Others**		1 (2.1)		1 (4.0)		0		
Total RT dose, cGy				5127.2 ± 1603.1		5374.4 ± 1741.1		4915.2 ± 1484.7		0.379
Mortality due to AE fistula				3 (6.3)		1 (4.0)		2 (8.7)		0.502
Progression pattern^a^		No progression		25 (52.1)		18 (72.0)		7 (30.4)		0.02
		Local progression		3 (6.3)		0		3 (13.0)		
		Regional progression		10 (20.8)		3 (12.0)		7 (30.4)		
		Distant metastasis		10 (20.8)		4 (16.0)		6 (26.1)		

Data are expressed as mean ± SD (range) or *n* (%)

*ASA* American Society of Anesthesiologists, *CAD* coronary artery disease, *CKD* chronic kidney disease, *COPD* chronic obstructive pulmonary disease, *DM* diabetes mellitus, *CRT* chemoradiation therapy, *cGy* centigray, *TEVAR* thoracic endovascular aortic repair, *RT* radiation therapy, *AE* aortoesophageal

^a^The definition of ‘progression pattern’ is described in the Methods

The mean follow-up period was 21.8 ± 27.8 months (overall range 1.0–143.5 months). The majority of patients were male (93.8%, 45/48) and the mean age was 58.8 ± 8.5 years. The most common American Society of Anesthesiologists (ASA) classifications at the time of diagnosis were 2 and 3 (50.0% and 39.6%, respectively). More than half of the included patients were smokers (83.3%, 40/48) and more than half were habitual drinkers of alcohol (66.7%, 32/48). Among the patients included in this study, 16.7% (8/45) had esophageal tumors located in the upper third of the esophagus, 39.6% (19/45) had tumors located in the middle third, and 43.8% (21/45) had tumors located in the lower third. The comorbidities and other demographic data are listed in Table 2. There were 8 cases of emergency TEVAR and 17 cases of prophylactic TEVAR. The median hospital stay after TEVAR was 12 days. There was no significant difference between the two groups of patients except that the postsurgical bleeding and blood stream infection complications were significantly higher in patients treated with emergency TEVAR (0 % vs. 50%, *p* = 0.001; and 5.9% vs. 50%, *p* = 0.022). No cerebral infarction or spinal cord injury occurred. The 30-day mortality rate after the TEVAR procedure was 8.0% (2/25), while the 90-day mortality rate was 16.0% (4/25).

**Table 2:** Perioperative outcome and detailed information about the TEVAR procedure

		Total [*n* = 25]	Prophylactic^a^ TEVAR [*n* = 17]	Non-prophylactic TEVAR [*n* = 8]	*p*-value
Device	Gore C-Tag	15 (60)	10 (58.8)	5 (62.5)	0.782
	Medtronic Valiant	8 (32)	6 (35.3)	2 (25.0)	
	Cook TX2 or Alpha	2 (8)	1 (5.9)	1 (12.5)	
Proximal landing zone	Zone 0	1 (4.0)	0	1 (12.5)	0.162
	Zone 1	0	0	0	
	Zone 2	5 (20.0)	3 (17.7)	2 (25.0)	
	Zone 3	9 (36.0)	5 (29.4)	4 (50.0)	
	Zone 4	10 (40.0)	9 (52.9)	1 (12.5)	
Diameter, mm	Proximal landing zone	30.9 ± 2.8	31.6 ± 3.2	29.3 ± 3.2	0.106
	Distal landing zone	28.2 ± 2.2	28.9 ± 2.7	26.9 ± 3.7	0.135
Length of coverage, mm		149.2 ± 14.9	153.5 ± 20.9	140.0 ± 25.6	0.173
Concomitant procedure	LSCA chimney stent	5 (20)	3 (17.7)	1 (12.5)	0.743
	LCCA chimney stent	1 (4)	0	1 (12.5)	0.136
	Innominate artery chimney	1 (4)	0	1 (12.5)	0.136
Hospital stay after TEVAR, days^b^		12.0	12.0	31.0	
Post-TEVAR blood stream infection		5 (20.0)	1 (5.9)	4 (50.0)	0.022
Post-TEVAR cerebral infarction		0			
Post-TEVAR spinal cord injury		0			
Post-TEVAR hemorrhage		4 (16.0)	0	4 (50.0)	0.001
Esophagectomy after TEVAR		10 (40.0)	8 (47.1)	2 (25.0)	0.293
CRT after TEVAR		14 (56.0)	11 (64.7)	3 (37.5)	0.201
Mortality due to AE fistula		1 (4.0)	0	1 (12.5)	0.136
30-day mortality		2 (8.0)	1 (5.9)	1 (12.5)	0.569
90-day mortality		4 (16.0)	2 (11.8)	2 (25.0)	0.399

Data are expressed as *n* (%) for continuous variables and mean ± standard deviation for categorical variables

*LSCA* left subclavian artery, *LCCA* left common carotid artery, CRT, chemoradiation therapy, *TEVAR* thoracic endovascular aortic repair, *CRT* chemoradiation therapy, *AE* aortoesophageal

^a^TEVAR performed under stable condition

^b^Expressed as median duration, days

The clinical profiles and perioperative outcomes related to esophagectomy are listed in electronic supplementary Tables 2 and 3. Esophagectomy was performed in 24/48 patients in this cohort, in which 75% (18/24) received a Mckeown procedure, while the remaining patients received an Ivor Lewis procedure (25%, 6/24). For those patients undergoing esophagectomy and pathological examination, 50.0% (12/24) were classified as pathological stage N0, 37.6% (9/24) were classified as N1, 8.4% (2/24) were classified as N2, and 8.4% (2/24) were classified as N3. Of the 48 patients, 19 (39.6%) received definite chemoradiation therapy without surgery. A higher total radiation dose was administered to those patients who received TEVAR than those who did not, although without a statistically significant difference (5374.4 ± 1741.1 vs. 4915.2 ± 1484.7, *p* = 0.379). A total of three patients died of AE fistulas; the first was a patient in the emergency TEVAR group who died of an esophagectomy-related AE fistula, and the second and third patients were in the non-TEVAR without surgery group and died due to AE fistulas that developed during the course of treatment.

**Table 3:** Univariate and multivariable analysis of correlation between clinical features and progression-free survival in esophageal cancer patients with aortic invasion

		Univariable			Multivariable		
		Progression HR	95% CI95% CI	*p*-value	Progression HR	95% CI	*p*-value
Age		0.975	0.929−–1.024	0.312			
Sex, male		0.308	0.069−–1.370	0.122			
ASA classification ≥3 ASA classification ≥3		1.313	0.588−2.936				
Smoking		0.354	0.140−–0.896	0.028	0.528	0.189–1.477	0.224
Clinical N positive Clinical N positive		0.875	0.258−2.965	0.830			
Subgroups^a^	A	1		1			
	B/C	2.845	0.855−–9.465	0.088	2.895	0.869−–9.641	0.083
	D	5.283	1.687−–16.546	0.004	4.371	1.333−–14.333	0.015

Subgroups: (A): Esophagectomy with TEVAR; (B/C): TEVAR or esophagectomy only; (D) No TEVAR or esophagectomy

*ASA* American Society of Anesthesiologists, *HR* hazard ratio, *CI* confidence interval, *TEVAR* thoracic endovascular aortic repair

^a^Trend test for correlation between disease-free survival and subgroups showed *p* = 0.003 in univariate analysis and *p* = 0.0.004 in multivariable analysis

## Overall and Progression-Free Survival Analysis

**Fig. 2:**
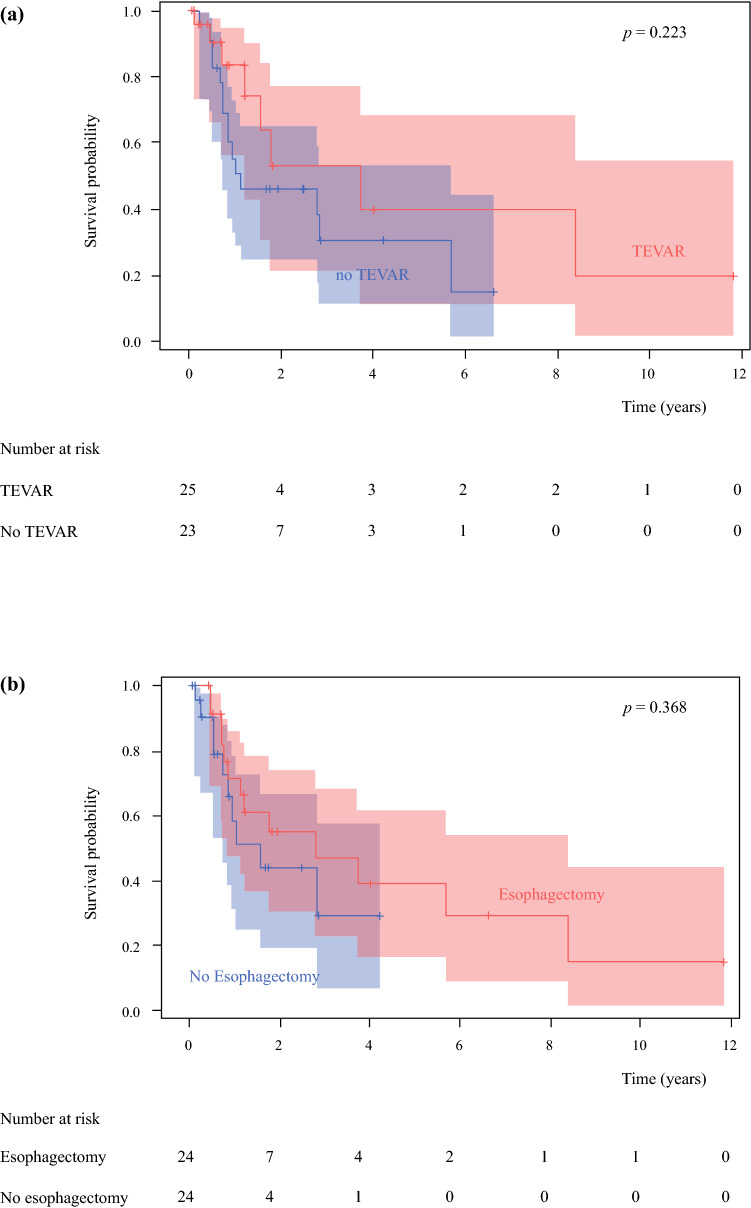
Overall survival curve between patients treated **a** with or without TEVAR *(p* = 0.223) and **b** with or without esophagectomy (*p* = 0.368). *TEVAR* thoracic endovascular aortic repair

The median OS of patients included in this study was 317.5 days (range 27–4319 days). After esophagectomy, the cumulative 1-year OS rate was 71.8% in our cohort, while the 1-year PFS rate was 65.9%. There was no statistical difference in the number of dissected lymph nodes in patients treated with and without TEVAR (22.3 ± 9.3 vs. 27.8 ± 13.5, *p* = 0.271). The median OS of patients treated with and without TEVAR was 260 days and 381 days, respectively, with no significant difference between the two groups according to Kaplan–Meier analysis with a log-rank test (*p* = 0.223) (Fig. 2a). Similar patterns of OS duration were observed in patients treated with or without esophagectomy, with a median OS of 443 days in patients undergoing esophagectomy and 248.5 days in those who did not (*p =* 0.368) (Fig. 2b).

**Fig. 3:**
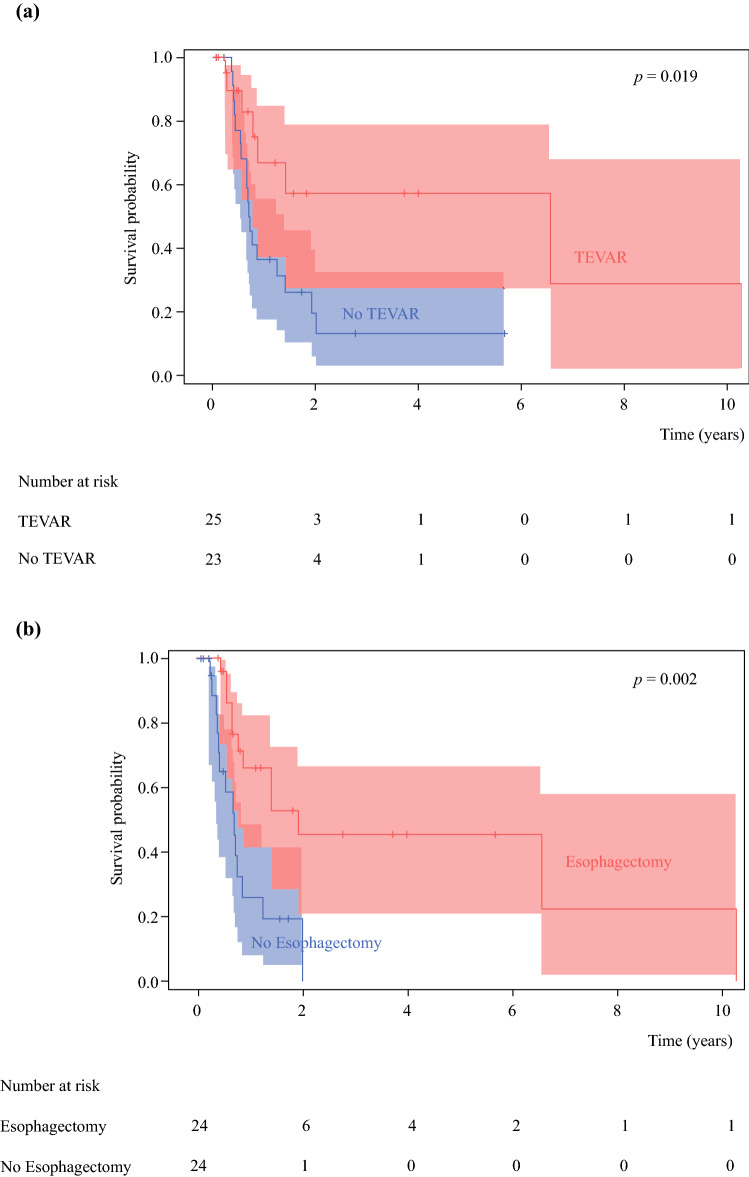
Progression-free survival curve between patients treated **a** with or without TEVAR (*p* = 0.019) and **b** with or without esophagectomy (*p* = 0.002). *TEVAR* thoracic endovascular aortic repair

The median PFS of the cohort was 251.5 days (range 27–3752 days). According to Kaplan–Meier analysis with the log rank test, PFS was statistically better for patients treated with TEVAR than those who were not treated with TEVAR (*p* = 0.019) (Fig. 3a). Esophagectomy was also associated with significantly better PFS (*p* = 0.002) (Fig. 3b).

In the subgroup analysis, we divided patients into four subgroups. Group A consisted of those patients who received both TEVAR and esophagectomy; Group B consisted of those who received esophagectomy but no TEVAR; Group C consisted of those who received TEVAR but no esophagectomy; and Group D consisted of those who did not receive either TEVER or esophagectomy. The detailed clinical profiles and oncological results for patients treated with or without TEVAR/esophagectomy are presented in electronic supplementary Table 1. No significant difference in OS was noted between the subgroups (*p* = 0.297) (electronic supplementary Fig. 1a); however, patients in group A did have significantly better PFS than patients in the other subgroups (*p* = 0.007) (electronic supplementary Fig. 1b). The univariate and multivariable analysis for factors influencing the disease PFS of patients are listed in Table 3. Those patients who did not receive both TEVAR and esophagectomy showed an incremental increase in hazard ratio (HR) for disease progression of 2.89 (95% confidence interval [CI] 0.8–9.96) and 4.37 (95% CI 1.33–14.33), respectively, with the absence of one of these procedures or both, according to multivariable analysis (*p* = 0.003 for trends of difference, under multivariable analysis) (Table 3).

## Discussion

Traditionally, esophageal cancer patients with evidence of invasion of the vertebral body, trachea, or aorta, i.e. clinical stage T4, are considered inoperable. According to the NCCN guidelines (version 4.2019), definite CCRT is preferred for these advanced diseases. However, instead of conservative therapy, some surgeons have adopted a more aggressive approach to esophageal cancer invading the aorta. Cong and colleagues reported 1- and 5-year OS rates of 80.9% and 21.3%, respectively, in patients who had esophageal cancer invading the aorta and received esophagectomy combined with aortic segment replacement,^15^ in comparison with a median survival of 10.6 months reported in the literature for such patients who received conservative treatment.^4^ This observation is consistent with the finding by other authors that radical surgical resection after chemoradiation can provide a chance of improved survival for selected patients with esophageal cancer invading an adjacent organ.^16^–^18^

TEVAR was first introduced in the 1990s for endovascular repair of aortic aneurysms in patients who were thought to be inappropriate for open repair.^19^ After success in the treatment of aortic aneurysm, the indications for TEVAR have expanded to include more complicated cases.^20^ To date, the feasibility and effectiveness of TEVAR for thoracic aortic aneurysms have generally been acceptable. In suitable patients, TEVAR even has results comparable with open repair.^21^ Given the advantages of TEVAR over open repair, such as minimal invasiveness, no aortic clamping, a lower incidence of spinal cord ischemia, and less use of anticoagulants, TEVAR is quickly becoming an ideal alternative procedure to traditional open surgical intervention for patients undergoing aortic intervention.^22^

The use of TEVAR in treating esophageal cancers invading the aorta is nonetheless currently not well-established and direct comparison between treatment outcomes with and without the application of TEVAR in these advanced esophageal cancers is lacking. In our study, there was a significant reduction of local progression of disease and improved progression-free disease in the TEVAR group when compared with the non-TEVAR groups. In addition, esophagectomy also provided a significantly better disease PFS than treatment without surgery. It is noteworthy that a complete resection can be achieved in 67% of patients (10/15) after TEVAR followed by CCRT and an incremental effect on the improvement in disease-free survival can be observed when combining these two factors, with an increased HR for disease progression attributed to the absence of any of these two factors. For patients who have received TEVAR, radical surgery with esophagectomy can be performed with a lower risk of aortic injury during the procedure. With the potential for improved local control, such disease may eventually be considered resectable, with appropriate validation of these findings. Although there was no statistical difference in OS between patients who did and did not undergo TEVAR, a trend of improvement could be seen in OS in the TEVAR group.

As a less invasive procedure than aortic resection, TEVAR can provide the possibility of curative resection of an invading tumor; however, there are also some concerns about complications associated with the procedure. Endoleak, the most common complication of TEVAR, has a reported incidence of 23.3–32.9% in patients with thoracic descending aortic disease undergoing TEVAR.^23^ Of the 25 patients treated with TEVAR in our study, only one patient had an endoleak and subsequently received re-intervention. Graft infection is another important complication in these esophageal cancer patients, with an estimated incidence rate of 0.5–5%, which is probably caused by the presence of tumor and perhaps necrotic tissue surrounding the stent.^24^ In our study, 8/25 patients experienced post-TEVAR blood stream infection, and one patient died from graft-related infection. Cerebral infarction and spinal cord injury are also severe complications after TEVAR, but no patient developed neurological deficit in our study. In our initial experience, TEVAR was only performed for those with suspected bleeding from an aortoesophageal fistula induced by tumor invasion. As shown in our results, patients receiving emergency TEVAR had a higher incidence of postoperative bleeding and systemic infection compared with those receiving prophylactic TEVAR. Half of the patients still experienced bleeding after emergency TEVAR compared with none in the prophylactic group. Given the higher risk of procedure-related complications, some surgeons have applied TEVAR for cases with esophageal cancer invading the aorta to prevent active or emergent bleeding in the aortic wall where the invasion occurs.^9^,^25^–^27^ This concept is similar to the treatment of aortic aneurysms, in which the surgical mortality rate associated with ruptured aneurysms is significantly higher than that of elective surgery.^28^,^29^ Because aortic bleeding occurs unexpectedly without specific preceding symptoms or signs, emergency TEVAR for hemostasis is often performed under poor clinical conditions, including shock status, higher susceptibility to infection, and multiple organ failure in patients, which are correlated with poor prognosis. Furthermore, performing TEVAR on these T4 esophageal cancer patients with aortic invasion not only prevents fatal bleeding but also bridges to increased safety in subsequent chemoradiotherapy or esophagectomy.^10^^,^^30^ Following the TEVAR procedure, none of the patients in the prophylactic group in our study, and only one patient in the emergency group undergoing esophagectomy, had bleeding from an AE fistulae, compared with two mortalities due to an AE fistulae in the non-TEVAR group treated without surgery.

We acknowledge several limitations of this study. For a retrospective study, there is inherent bias including the timing of the TEVAR procedure and extent of esophagectomy and lymph node dissection. Due to the long time span of this study, the treatment strategies and outcomes were not equivalent in the earlier and later periods. In our hospital, prophylactic TEVAR procedures were performed mostly after 2016, which perhaps provided better survival.

We also acknowledge the limitation of statistical power under such a small patient population, although it is currently the largest cohort in the literature to apply TEVAR in advanced esophageal cancer invading the aorta. All TEVAR procedures and surgeries for esophageal cancers were performed by a single surgical team, thus ensuring consistency of the treatment methods. Moreover, this is the only study that provides the results of direct comparison between treatment with and without TEVAR in esophageal cancers invading the aorta. We reported not only the perioperative outcome after using TEVAR in treating such esophageal cancers but also comprehensive long-term OS and PFS data. It is noteworthy that even under such limited statistical power, the difference in PFS among subgroups of patients can be observed under the model of multivariable analysis. It is worthwhile conducting multi-institutional prospective studies in the future to further investigate whether the intervention with TEVAR can make a difference in survival for such patients.

## Conclusion

TEVAR in conjunction with esophagectomy provides better PFS in patients with esophageal cancers and aortic invasion. TEVAR performed in prophylaxis for such patients is feasible and can facilitate subsequent curative resection for advanced esophageal cancers with aortic invasion. When used with the TEVAR procedure, esophagectomy can be a treatment choice and can provide better PFS for those advanced esophageal cancers that were once considered to be unresectable.

## Supplementary Information

Below is the link to the electronic supplementary material.

Supplementary file1 (DOCX 131 kb)
