# Supplementary material for: The Long-Term Clinical Impact of Thoracic Endovascular Aortic Repair (TEVAR) for Advanced Esophageal Cancer Invading Aorta
Source: Ann Surg Oncol. 2021 Jun 3;28(13):8374–84. doi: 10.1245/s10434-021-10081-3 (PMC8591004; doi:10.1245/s10434-021-10081-3)

Supplementary Table 1. Clinical information about the enrolled patients

|  |  | Total | Subgroups | | | |
| --- | --- | --- | --- | --- | --- | --- |
|  |  | **(n=48)** | **A (n=15)** | **B (n=9)** | **C (n=10)** | **D (n=14)** |
| Sex (male) |  | 45 (93.8%) | 15(100%) | 8(88.9%) | 10(100%) | 12(85.7%) |
| Age (y/o) |  | 58.8±8.5 | 60.0±6.3 | 59.0±10.9 | 60.6±11.5 | 56.1±6.5 |
| ASA classification | 1 | 0 | 0 | 0 | 0 | 0 |
|  | 2 | 24 (50%) | 10(66.7%) | 4(44.4%) | 6(60.0%) | 4(28.6%) |
|  | 3 | 19 (39.6%) | 4(26.7%) | 4(44.4%) | 3(30.0%) | 8(57.1%) |
|  | 4 | 5 (10.4%) | 1(6.7%) | 1(11.1%) | 1(10.0%) | 2(14.3%) |
|  | 5 | 0 | 0 | 0 | 0 | 0 |
| Smoking |  | 40 (83.3%) | 14(93.3%) | 8(88.9%) | 6(60.0%) | 12(85.7%) |
| Drinking |  | 32 (66.7%) | 9(60.0%) | 8(88.9%) | 5(50.0%) | 10(71.4%) |
| Betelnut chewing |  | 16 (33.3%) | 5(33.3%) | 3(33.3%) | 2(20.0%) | 6(42.9%) |
| Comorbidity | CAD | 2 (4.2%) | 1(6.7%) | 1(11.1%) | 0 | 0 |
|  | Liver cirrhosis | 2 (4.2%) | 0 | 0 | 2(20.0%) | 0 |
|  | CKD | 1 (2.1%) | 0 | 0 | 1(10.0%) | 0 |
|  | COPD | 2 (4.2%) | 0 | 1(11.1%) | 0 | 1(7.1%) |
|  | DM | 3 (6.3%) | 1(6.7%) | 0 | 2(20.0%) | 0 |
|  | Heart failure | 1 (2.1%) | 0 | 0 | 0 | 1(7.1%) |
|  | Hypertension | 11 (22.9%) | 6(40%) | 2(22.2%) | 3(30.0%) | 0 |
|  | Hyperlipidemia | 1 (2.1%) | 0 | 0 | 1(10.0%) | 0 |
|  | Others | 16 (33.3%) | 5(33.3%) | 2(22.2%) | 5(50.0%) | 4(28.6%) |
| Tumor location | Upper | 8 (16.7%) | 4(26.7%) | 1(11.1%) | 0 | 3(21.4%) |
|  | Middle | 19 (39.6%) | 2(13.3%) | 2(22.2%) | 7(70.0%) | 8(57.1%) |
|  | Lower | 21 (43.8%) | 9(60.0%) | 6(66.7%) | 3(30.0%) | 3(21.4%) |
| Pathological N stage | pN0 | 12(25.0%) | 7(46.7%) | 5(55.6%) | 0 | 0 |
|  | pN1 | 9(18.8%) | 5(33.3%) | 4(44.4%) | 0 | 0 |
|  | pN2 | 2(4.2%) | 2(13.3%) | 0 | 0 | 0 |
|  | pN3 | 2(4.2%) | 1(6.7%) | 0 | 1(10.0%) | 0 |
|  | cNx  (no surgery) | 23(47.9%) | 0 | 0 | 9(90.0%) | 14(100.0%) |
| Esophagectomy and reconstruction | No surgery | 24(50.0%) | 0 | 0 | 10 (100.0%) | 14 (100.0%) |
|  | Tri-incision | 18(37.5%) | 5 (33.3%) | 9 (100.0%) | 0 | 0 |
|  | Ivor Lewis | 6(12.5%) | 10 (66.7%) | 0 | 0 | 0 |
| CRT | No | 1(2.1%) | 0 | 0 | 1(10.0%) | 0 |
|  | Definite | 19(39.6%) | 0 | 0 | 6(60.0%) | 13(92.9%) |
|  | Neoadjuvant | 19(39.6%) | 9(60.0%) | 7(77.8%) | 3(30.0%) | 0 |
|  | Adjuvant | 3(6.3%) | 2(13.3%) | 0 | 0 | 1(7.1%) |
|  | Neoadjuvant + Adjuvant | 6(12.5%) | 4(26.7%) | 2(22.2%) | 0 | 0 |
| Total RT dose (cGy) |  | 5127.2  ±1603.1 | 5761.5  ±1430.4 | 4573.3  ±1252.6 | 4368.0  ±2234.3 | 5171.7  ±1643.0 |
| Mortality due to AE fistula |  | 3(6.3%) | 1(6.7%) | 0 | 0 | 2(14.3%) |
| Progression pattern* | No progression | 25(52.1%) | 9(60.0%) | 5(55.6%) | 9(90.0%) | 2(14.3%) |
|  | Local progression | 3(6.3%) | 0 | 0 | 0 | 3(21.4%) |
|  | Regional progression | 10(20.8%) | 3(20.0%) | 3(33.3%) | 0 | 4(28.6%) |
|  | Distant metastasis | 10(20.8%) | 3(20.0%) | 1(11.1%) | 1(10.0%) | 5(35.7%) |

Data are presented as mean ± SD (range) or number (%).

Abbreviations: ASA classification, American Society of Anesthesiologists Classification; CAD, coronary artery disease; CKD, chronic kidney disease; COPD, chronic obstructive pulmonary disease; DM, diabetes mellitus; CRT, chemoradiation therapy; cGy, centigray.

*The definition of progression pattern is described in Methods. Subgroups: (A): Esophagectomy with TEVAR ; (B): Esophaectomy only; (C): TEVAR only, (D): None of TEVAR or esophagectomy

# Supplementary Table 2. Comparison of clinical characteristics and outcomes in patients treated with and without esophagectomy

|  |  | Total (n=48) | Esophagectomy (n=24) | No esophagectomy (n=24) | *p* value |
| --- | --- | --- | --- | --- | --- |
| **Sex (male)** |  | 3(6.2%) | 2(8.3%) | 1(4.2%) | 1 |
| **Age** |  | 58.8 ± 8.5 | 58.0 ± 9.0 | 59.6 ± 8.1 | 0.897 |
| **ASA classification** | 1 | 0 |  |  | 0.585 |
|  | 2 | 24(50.0%) | 10(41.7%) | 14(58.3%) |  |
|  | 3 | 19(39.6%) | 11(45.8%) | 8(33.3%) |  |
|  | 4 | 5(10.4%) | 3(12.5%) | 2(8.3%) |  |
| **Smoking** |  | 40(83.3%) | 18(75.0%) | 22(91.7%) | 0.245 |
| **Drinking** |  | 32(66.7%) | 15(62.5%) | 17(70.8%) | 0.54 |
| **Betel nuts** |  | 16(33.3%) | 8(33.3%) | 8(33.3%) | 1 |
| **Comorbidity** | CAD | 2(4.2%) | 0 | 2(8.3%) | 0.489 |
|  | Liver cirrhosis | 2(4.2%) | 2(8.3%) | 0 | 0.489 |
|  | CKD | 1(2.1%) | 1(4.2%) | 0 | 0.585 |
|  | COPD | 2(4.2%) | 1(4.2%) | 1(4.2%) | 1 |
|  | DM | 3(6.2%) | 2(8.3%) | 1(4.2%) | 1 |
|  | Heart failure | 1(2.1%) | 1(4.2%) | 0 | 1 |
|  | Hypertension | 11(22.9%) | 3(12.5%) | 8(33.3%) | 0.168 |
|  | Hyperlipidemia | 1(2.1%) | 1(4.2%) | 0 | 1 |
|  | Others | 16(33.3%) | 9(37.5%) | 7(29.2%) | 0.54 |
| **Tumor location** | Upper | 8(16.7%) | 3(12.5%) | 5(20.8%) | 0.003 |
|  | Middle | 19(39.6%) | 15(62.5%) | 4(16.7%) |  |
|  | Lower | 21(43.8%) | 6(25.0%) | 15(62.5%) |  |
| **Pathological N stage** | P or c N0 | 12(25.0%) | 0 | 12(50.0%) | <0.001 |
|  | p or c N1 | 9(18.8%) | 0 | 9(37.5%) |  |
|  | p or c N2 | 2(4.2%) | 0 | 2(8.3%) |  |
|  | p or c N3 | 2(4.2%) | 1(4.2%) | 1(4.2%) |  |
|  | no surgery | 23(47.9%) | 23(95.8%) | 0 |  |
| **TEVAR** |  | 25(52.1%) | 10(41.7%) | 15(62.5%) | 0.149 |
| **CRT** | No | 1(2.1%) | 1(4.2%) | 0 | <0.001 |
|  | Definite | 19(39.6%) | 19(79.2%) | 0 |  |
|  | Neoadjuvant | 19(39.6%) | 3(12.5%) | 16(66.7%) |  |
|  | Adjuvant | 3(6.2%) | 1(4.2%) | 2(8.3%) |  |
|  | Neoadjuvant + Adjuvant | 6(12.5%) | 0 | 6(25.0%) |  |
| **Total RT dose(cGy)** |  | 5217.2±1603.1 | 4935.3±1801.7 | 5275.5±1457.5 | 0.978 |
| **Death due to AE fistula** |  | 3(6.3%) | 1(4.2%) | 2(8.3%) | 0.551 |
| **Progression pattern*** | No progression | 25(52.1%) | 11(45.8%) | 14(58.3%) | 0.298 |
|  | Local progression | 3(6.2%) | 3(12.5%) | 0 |  |
|  | Regional progression | 10(20.8%) | 4(16.7%) | 6(25.0%) |  |
|  | Distant metastasis | 10(20.8%) | 6(25.0%) | 4(16.7%) |  |

Data are presented as mean ± SD (range) or number (%).

Abbreviations: ASA classification, American Society of Anesthesiologists Classification; CAD, coronary artery disease; CKD, chronic kidney disease; COPD, chronic obstructive pulmonary disease; DM, diabetes mellitus; CRT, chemoradiation therapy; cGy, centigray.

*The definition of progression pattern is described in Methods

Supplementary Table 3. Perioperative outcome in patients who underwent esophagectomy and reconstruction

|  | Esophagectomy  (n=24) |
| --- | --- |
| Margin free | 19 (79.2%) |
| LN dissected (station number) | 6.3±2.0 |
| LN dissected (total number) | 24.5±11.2 |
| 30-day mortality | 2 (8.3%) |
| 90-day mortality | 4 (16.7%) |
| Adjuvant RT | 9 (37.5%) |
| Total RT dose | 5275.5±1457.5 |
| 1-y OS rate | 71.8% |
| 1-y PFS rate | 65.9% |

Data are presented as mean ± SD (range) or number (%).

Abbreviations: RT, radiation therapy; cGy, centigray

Supplementary Figure 1. The overall and progression-free survival curves among the 3 groups of patients

A


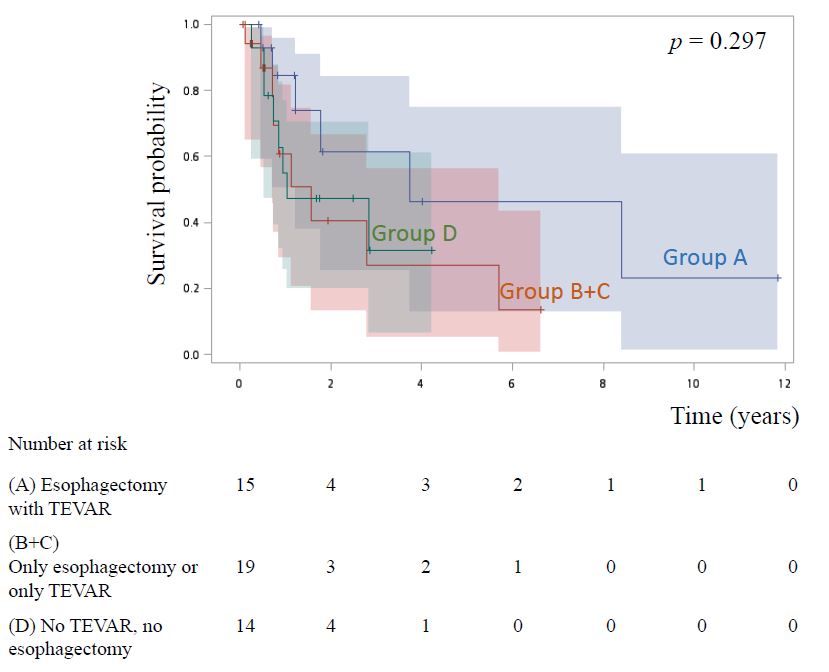


B


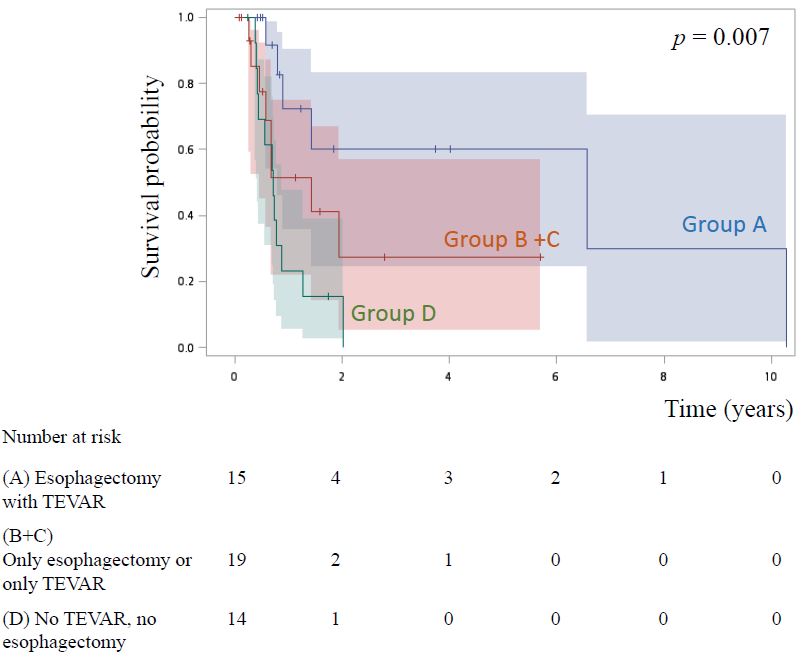

Supplement: Supplementary file 1 — Supplementary file1 (DOCX 131 kb) [file 10434_2021_10081_MOESM1_ESM.docx]
